# Stitch-Less Lithography Empowered by Multi-Dimensional Holography

**DOI:** 10.3390/nano16110692

**Published:** 2026-06-01

**Authors:** Hsin-Hui Huang, Haoran Mu, Eulalia Puig Vilardell, Vijayakumar Anand, Darius Gailevičius, Saulius Juodkazis

**Affiliations:** 1 Optical Sciences Centre, Swinburne University of Technology, Melbourne, VIC 3122, Australia; haoranmu@swin.edu.au (H.M.); vijayakumar.anand@ut.ee (V.A.); 2 Australian Research Council (ARC), Industrial Transformation Training Centre in Surface Engineering for Advanced Materials (SEAM), Swinburne University of Technology, Melbourne, VIC 3122, Australia; 3 Melbourne Centre for Nanofabrication (MCN), 151 Wellington Road, Melbourne, VIC 3168, Australia; 4 Laser Research Center, Physics Faculty, Vilnius University, Saulėtekio Ave. 10, 10223 Vilnius, Lithuania; eulalia.puig@ff.stud.vu.lt (E.P.V.); darius.gailevicius@ff.vu.lt (D.G.); 5 Institute of Physics, University of Tartu, W. Ostwaldi 1, 50411 Tartu, Estonia; 6 World Research Hub (WRH), School of Materials and Chemical Technology, Institute of Science Tokyo, 2-12-1, Ookayama, Meguro-ku, Tokyo 152-8550, Japan

**Keywords:** stitching-less lithography, resolution, feature size, threshold effect, diffraction limit, microfabrication, 3D

## Abstract

Trends in Micro- and Nano-Lithography required for future development of large area applications ranging from high-packing-density electronics to solar cells are surveyed and outlined. Strategies to use direct laser writing to define etch masks over large areas by: (i) fixed beam moving stage and (ii) moving beam moving stage approaches are presented. The extension of planar 2D and stacked 2D (or 2.5D) fabrication methods into 3D micro- and nano-fabrication is discussed. One of the essential future characteristics of 3D nanolithography is real-time feedback capability. This can be realised via inherent 3D-capable holography, which bridges lithographic exposure control, wavefront sensing, and adaptive feedback, providing a pathway to stitch-free, large-area 3D patterning. The future of micro-fabrication is expected to evolve via highly specialised 3D architecture design and reduction in post-processing steps.

## 1. E/O-Moore’s Laws

**Figure 1:**
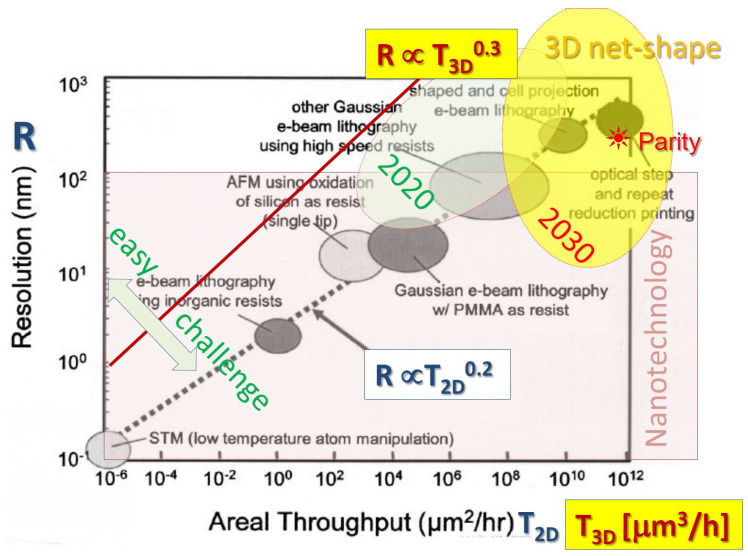
Our established 3D printing dependence of resolution *R* vs. throughput $T_{3D}$ is based on Tennant’s scaling [15], to be further facilitated by Moore’s law for average power fs-laser we revealed [4]. The recent (2020) and future (2030) throughput and resolution capability ranges are shown. Conventional electrical discharge machining (EDM) achieves 3 mm^3^/min material removal rate. The Parity point using fs-laser fabrication was reached in 2016 [16], when the material removal rate by laser and by current spark (mechanical)-machining become equal. Mesoscale resolution from nm-to-mm is the aim based on high-energy density fabrication.

The (electronics) e-Moore’s law of transistor number doubling every two years [1,2,3], which is based on the development of planar two-dimensional (2D) nanolithography (Figure 1), is the driving force for the growth of the closely interconnected fields of computers, telecommunications, fibre-optics, mobile phones, and, more recently, artificial intelligence (AI). The (optical) o-Moore’s law shows the same tendency of doubling the number of photons every two years (and hence average laser power) for ultra-short pulsed lasers [4,5,6]. This tendency is clear only for the past 25 years [7]. Arguably, it is driven by new applications of ultra-short laser machining [8,9], which match the challenge for higher average power requirements by the advancement of laser sources when more power is extracted from the excited crystal in the cavity [10,11,12,13].

**Figure 2:**
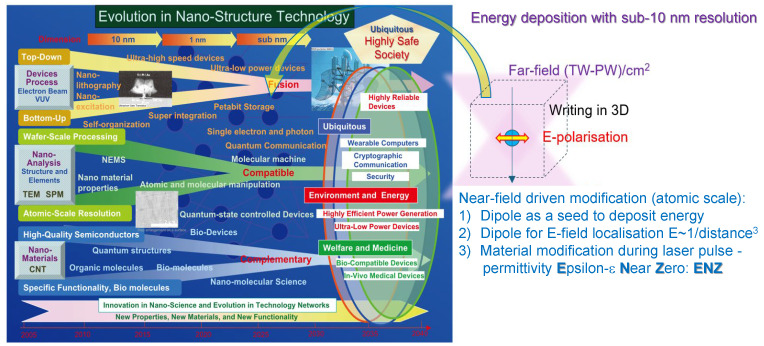
The “fusion era” of previously exclusive approaches is expected to start from 2025 onward. This roadmap, developed by the Japan Society of Applied Physics (JSAP)—a leading knowledge economy—strategically addresses societal needs [17] (copyright publication 2013 The Japan Society of Applied Physics). The right-side inset shows a dipole nano-seed as a 2D/3D laser writing tool. The intensity of the dipole source is localised as $I\propto r^{6}$, where *r* is the polar coordinate (radius of point-like energy deposition). The most efficient energy deposition is near the dielectric breakdown, which is defined when permittivity $\varepsilon =n^{2}-\kappa^{2}=0$ defined by the complex refractive index $\tilde{n}=n+i\kappa$. This is the ENZ state of material.

The fundamental difference between the two laws, e-Moore’s and o-Moore’s laws, is that the latter powers applications of direct laser write and sequential material processing (machining) [4,14], while e-Moore’s law’s success is due to its parallel-processing nature. In this perspective, promising approaches for the development of large-area direct write applications are overviewed, with arguments presented for when direct write (sequential mode) could challenge parallel-mode lithography. In these areas of technology development, the potential for real industrial application is greatest. Now is the right time to ask questions about promising directions and anticipate the most efficient uptake of laser technologies. This tension between sequential and parallel approaches is not only technical, but also reflects a broader industrial logic: performance leads, cost follows. This is the “Better Before Cheaper” principle at the global trend of the “fusion-era” (Figure 2).

## 2. BBC—Better Before Cheaper

The motivation for this perspective is to identify which emerging fabrication technologies can deliver meaningful gains in performance and functionality, based on their technical simplicity or complexity, flexibility across different material systems, ability to operate over wide spatial and temporal scales, and overall technological maturity.

Direct laser writing and machining, compared to extreme ultraviolet (EUV), X-ray, e-beam, or ion-beam tools, have historically been adopted first in application domains where flexibility, rapid iteration, and material compatibility outweigh pure throughput. This dynamic reflects a broader “Better Before Cheaper” (BBC) principle [18]. It refers to the observation that higher margins from differentiation contribute more to the return on investment than efficiency gains from cost leadership [19]. In the current context, this relates to device architectures, substrates, and functional requirements that evolve rapidly; performance and adaptability must advance before cost or large-scale productivity becomes decisive. A decade ago, ultrashort-pulse laser fabrication had already enabled optical memories with densities beyond 1 Tbit/cm^3^, waveguide-based optical information processing structures, 3D photonic crystals, micro-mechanical devices, and components for optical quantum systems [15,20,21,22,23]. These achievements underscored that early adoption was driven by function-first demand: researchers needed access to programmable, maskless, three-dimensional fabrication long before parallel lithography infrastructures could accommodate such geometries.

Today, in 2026, this trajectory has widened considerably, with applications ranging from high-packing-density electronics to solar cells increasingly relying on heterogeneous material stacks, extended substrate sizes, and device architectures that combine nanometre-scale features with micrometre- to millimetre-scale structures [24,25]. These emerging domains—including 3D integrated photonics, metasurface-encoded optics, quantum photonics, and microfluidic–opto-mechanical systems—require fabrication strategies capable of tolerating material diversity and supporting rapid design variation within a single multi-scale workflow [15,26,27,28,29,30,31]. In this context, lithographic strategies that emphasise flexibility, programmability, and multi-scale capability become particularly attractive. Examples illustrating how the flexibility of direct-writing approaches spans from fundamental material limits to full device-scale architectures can be found across diverse material platforms and applications. In the domain of 2D materials, which have emerged as an especially powerful platform for miniaturised optoelectronic and photonic devices, direct laser writing (via femtosecond laser scanning or scribing) has demonstrated angstrom-level removal depths and single-layer precision. This capability enables the thinning of graphene films to specific layer counts [32], as well as the fabrication of ultra-thin flat lenses through precise patterning of transition-metal dichalcogenides (TMDs) [33]. Moving from the control of vertical layer thickness to the pursuit of lateral resolution, direct super-resolution writing on GaAs has achieved lateral feature sizes as small as 32 nm, approximately one-tenth the diameter of the focused laser spot [34]. This was realised by selectively utilising the central region of the laser spot that exceeds the ablation threshold, enabling the fabrication of periodic nanostructures such as gratings and nano-holes. Such precision is essential for site-controlled quantum-dot growth and high-performance anti-reflection coatings. Resist-free femtosecond laser patterning of graphene, MoS_2_, and PtSe_2_ has been demonstrated with sub-100 nm features using a commercial two-photon printer clearing a 200 μm × 200 μm area under 3 s [35]. On the opposite scale, direct writing inside bulk glass has produced millimetre-scale plasmonic nanostructure arrays via near-field interaction, yielding structural colour and linear dichroism responses buried within the glass [36]. Together, these examples demonstrate that direct-writing techniques enable fabrication from the atomic-layer level to the architectural scales relevant for next-generation quantum and optoelectronic technologies.

**Figure 3:**
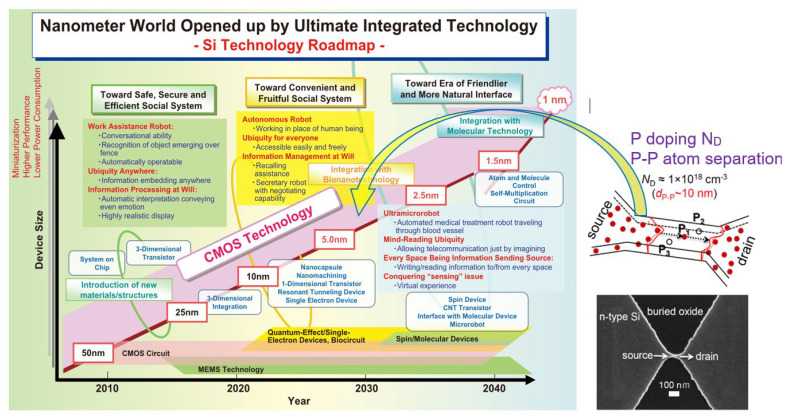
The potential of Si due to established CMOS technology [17] (copyright publication 2013 The Japan Society of Applied Physics). A Si-based CMOS-compatible approach for laser patterning of silicon to enable hyper-doping will allow single-electron transistors (SETs) to operate at room conditions. The Si solar cell technology will surpass the current ray-optics Lambertian light-trapping limit using large-area ∼1 m^2^ sub-micrometre patterning via femtosecond laser direct writing. The right-side inset illustrates an SET where a p-dopant atom (a quantum dot) controls the drain current. Hyper-doping at a concentration of $10^{20}$ cm^−3^ is required and could be achieved by fs-pulse-triggered micro-explosion. At $10^{18}$ cm^−3^, p-to-p dopant separation is ∼10 nm; courtesy of Prof. D. Moraru.

Manufacturing has traditionally progressed in 7–10-year renovation cycles, prioritising reliability, tool longevity, and return on capital investment [15]. However, the industrial landscape has shifted: 3D device architectures, backside routing, advanced packaging, and yield-driven design now dominate lithographic requirements [24]. While resolution remains essential (Figure 3), system performance increasingly depends on depth-of-focus, overlay stability across warped or large substrates, and suppression of stochastic patterning errors such as line edge roughness (LER), or partially stochastic errors such as edge placement error (EPE) and critical dimension uniformity (CDU) [24]. Packaging lithography in particular demands a large depth of field (DOF) and precise alignment on non-flat surfaces, conditions for which mask-based projection tools are not inherently optimised [24]. These realities reinforce the BBC logic: industrial scaling now values precision, adaptiveness, and local correction as highly as raw throughput. Direct writing, being maskless and reconfigurable at the level of individual features, is uniquely positioned to supply “better” fabrication capabilities before high-volume processes can catch up.

This shift is supported by substantial technical advances in fs-laser fabrication. New generations of reliable MHz-to-GHz-class ultrafast sources, improved control of sub-100 nm feature sizes, and multi-NA mesoscale strategies (MBMS) enable structures spanning up to five orders of magnitude in scale [15]. Meanwhile, the broader lithography landscape, such as optical, EUV, e-beam, X-ray, and ion beam, shows clearer separation of strengths and limits [37]. Optical lithography remains diffraction-constrained and mask-dependent; EUV faces challenges related to LER, stochastic defects, resist chemistry, mask contamination, and high-vacuum infrastructure; e-beam maintains the finest resolution but remains throughput-limited; X-ray and ion-beam techniques use shorter wavelengths but carry burdens of mask deformation, vacuum requirements, or substrate damage [37].

Direct laser writing complements these methods by offering rapid prototyping, geometry freedom, and material diversity, often used to validate architectures before transferring stable parts of the flow to cheaper, higher-throughput projection lithography. The easiest way to imagine it is to consider master template fabrication using direct laser writing for nanoimprint lithography (NIL) [38,39]. Recent demonstrations prominently feature this BBC-workflow, in which complex micro-optics [40], photonic resonators [41,42], and plasmonic waveguides [43] are initially mastered via multiphoton lithography and subsequently scaled for mass production via soft NIL [38]. Most recently, this approach was extended to 3D achromatic metalenses for full-colour near-eye displays, with TPL-defined nanostructures replicated at scale via NIL [44]. It provided a rapid model-to-prototype pathway [45,46].

The emergence of 3D devices, backside metallisation, and chiplet-based integration requires fabrication strategies that provide localised precision, adaptive overlay control, and high depth-of-focus performance on non-planar or heterogeneous substrates [24]. These architectures intensify the demand for maskless, feedback-enabled direct writing, especially in stitching-critical or multi-material regions where traditional projection lithography struggles. Advanced packaging adds further constraints, as large substrates require a high DOF and tight overlay, while exposure methods that provide both high DOF and small pitches tend to be slow and expensive [24]. When combined with computational imaging and holography, femtosecond-laser-based direct writing can maintain accuracy over large areas, compensate for local topography, and suppress stochastic variations during fabrication. In this sense, BBC becomes a practical deployment strategy: rely first on methods that provide superior control and flexibility, then transition defined modules into high-throughput or lower-cost technologies once design and material specifications stabilise.

## 3. Stitching

One of the major challenges in large-area lithography is compensating for and reducing stitching errors when high-precision write fields must be seamlessly connected [47,48]. Another challenge is making high-resolution lithography masks (transmission or reflection) using electron beam lithography (EBL) [49,50]. Since such masks are made once and used for multiple (parallel) exposures, the efficiency of the approach is not bottlenecked [51]. For direct laser write (sequential) lithography, the bottleneck in producing the final pattern has to be addressed [52].

In EBL, the fixed beam moving stage (FBMS) was introduced, where a simple raster scan on a small scale is controlled by e-beam optics while e-resist is exposed on the substrate by moving the stage [53]. Raith EBL showed 10-nm-wide and 10-cm-long lines defined in the mask by EBL [54]. This technology was essential to progress in the last decade (or two) for micro-electronics due to the larger flexibility of available patterning compared with more specialised and faster pattern projection EBL tools [54,55]. This “democratisation” of EBL tools (also less capital investment) is well aligned with the popularity of 3D printing, which started as a pure prototyping method but has become a driving force in 3D fabrication in a very broad range of fields. The popularity of 3D printing is also based on the low cost of capital investment required, as well as the flexibility to change patterns and materials. It is noteworthy that recent direct write laser lithography tools based on laser diodes, e.g., PICOMASTER series, Raith, continue on stitch-less FBMS paradigm and can combine regions patterned at different resolutions R∝1/NA by choice of objective lens with different numerical aperture NA.

**Figure 4:**
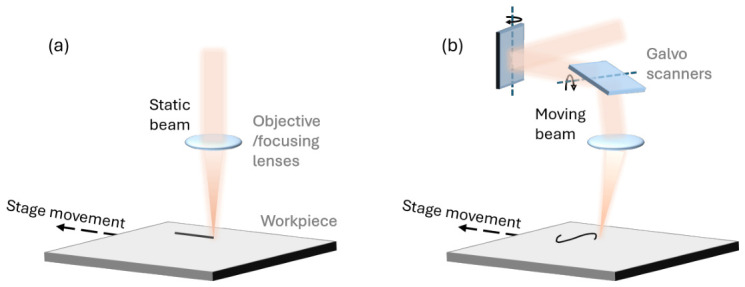
(**a**) Fixed beam moving stage (FBMS) and (**b**) moving beam moving stage (MBMS) operational differences.

In 3D laser lithography by direct writing, patterning areas larger than the available exposure or writing field of the tool was initially accomplished by separating the target layout into segments, sequentially writing and merging them into a continuous pattern [56]. Because adjacent fields must be precisely aligned and overlapped at their boundaries, any positional or geometric discrepancy in the stage or optical/scanning motion results in discontinuities, commonly termed stitching errors [56]. Stitching errors are therefore an intrinsic consequence of large-area patterning using mosaicked writing fields, which must be minimised to maintain pattern fidelity and device performance [57,58]. In practice, both minimisation and compensation strategies are used to address them [52]. A more flexible system that FBMS developed for EBL was made for 3D laser writing, which can be called moving beam moving stage (MBMS) [59], Figure 4. This MBMS 3D laser printing is demonstrated with different parts of the structure with resolution and feature sizes from ∼0.1 μm to ∼10 mm, hence, spanning five orders of magnitude at the mesoscale [59]. This is achieved by a change in the numerical aperture NA of focusing optics as well as pulse energy and dose (via a scanning protocol) [57,60]. Changing the objective lens along the same optical axis does not change the lateral focal position by more than (λ/NA)/20, which is suitable for most demanding optical applications [61].

Lithographic definition of large masks over areas with cross sections tens-of-cm is another challenge relevant to stitching errors as well as for mask adhesion to the substrate. Plasma etching is preferable to wet etching using masks for defining nano-sized 3D surface structures, since capillary forces during rinsing and drying of the fabricated structures can induce mechanical failure and adhesion [62]. Critical point drying (CPD) is not always available/applicable and is an additional complication of technological processes. Plasma etching has another virtue: it preserves the designed pattern even in the case of under-cut etch (wet etching can damage the pattern once under-cut etch occurs below the mask) [63,64]. The downside of plasma etch is the affected semiconductor surface with tens-of-nanometres into the sub-surface region, and passivation by wet or dry processing should be applied after the etch [65,66].

For large-area etch mask definition by lithography down to sub-micrometre structures, fs-laser direct write in tens-of-nm of alumina Al_2_O_3_ was proposed [67]. Ablation of dielectric nano-film opens a 200–300 nm hole for plasma etch [68]. Plasma etching removes the laser-affected region and forms an etched pit in the semiconductor/substrate [25,67]. The pattern of surface micro-pits for light trapping on the surface of a solar cell was made by this method [69]. This technology allows interference to be harnessed and can lead to 30% efficient Si solar cells and surpass the Lambertian light trapping limit all modern solar cells have as the limit [25,70]. By intentionally introducing non-ablated sites into a large area pattern [69], the mask area is defined and can withstand undercut etch. This method of mask definition is compatible with the MBMS approach and can be scalable up to solar panel areas of ∼1 m^2^ [68]. One more advantage of direct write on large areas is the possibility to track the surface in real time, while the projection of a large mask in parallel lithography mode is more sensitive to good planar alignment [61,71].

Mitigation of stitching errors, whether through MBMS writing, adaptive stitching algorithms, or holographic parallel exposure, addresses only the planar, 2D dimension of the control problem. The deeper challenge is that femtosecond direct write is not a planar process: it creates three-dimensional (3D) distributions of modified material states inside a volume, and tracking, characterising, and correcting that volume in real time requires a fundamentally different approach.

## 4. 3D Challenge

The integration of multilayered electronic chips, optical interconnects, and 3D connector lines increasingly requires fabrication methods that can operate inside complex opto-electronic assemblies rather than only on planar surfaces (see roadmaps in Figure 2 and Figure 3). Ultra-short-pulse laser machining is well suited to this task because it can deliver highly localised energy deposition with minimal mechanical contact and can, in principle, access buried or non-planar regions. The central difficulty, however, is no longer only how to write a pattern, but how to navigate and control the laser–material interaction in three dimensions.

In conventional planar lithography, the lateral position is usually constrained by stage encoders, scanner calibration, or interferometric positioning. By contrast, the axial coordinate, usually denoted as *Z*, is more difficult to stabilise because the focusing optics must remain suspended above the sample while the surface may be curved, tilted, thermally drifting, or optically heterogeneous. For delicate, transparent, multilayered, or partially processed substrates, contact-based height sensing is generally unsuitable; therefore, real-time optical focus tracking becomes essential. The practical requirement is stringent: the system must determine the position of the focal volume with minimal latency, preferably at nanoscale precision, while the sample and the written structure evolve during processing.

Several optical autofocus strategies have been developed for this purpose, including astigmatic focus-error detection, confocal reflectometry, and low-coherence interferometric sensing. Astigmatic detection is one of the most widely deployed focus-error sensing architectures [72,73,74,75]. In this method, the reflected tracking beam passes through a cylindrical lens before reaching a quadrant photodiode, so that axial displacement is converted into a change in the ellipticity and orientation of the detected spot [72,76]. Its main advantage is speed: the focus-error signal can be generated with little or no computational delay, making the method attractive for high-bandwidth surface tracking [76]. Its limitation is that the signal is sensitive to sample tilt, local reflectivity, diffraction from patterned surfaces, and parasitic reflections from transparent layers [73,76,77,78,79].

Confocal methods provide a more selective axial response and are therefore useful for structured, reflective, or multilayered substrates [80,81,82]. They rely on spatial filtering: light from the focal plane is transmitted through a pinhole or equivalent detection aperture, while out-of-focus light is strongly rejected [83,84,85]. By detecting the axial position of maximum returned intensity, the system identifies the best focus [86,87]. The trade-off is that many confocal implementations require axial scanning, source modulation, or objective dithering to locate the peak, which introduces latency relative to purely astigmatic detection [87,88].

When absolute distance measurement, transparent-layer metrology, or robust operation over difficult topographies is required, low-coherence interferometry becomes the most powerful option [89,90,91]. These systems use broadband or swept-source illumination to compare the optical path length from the sample with that from a reference arm [90,92,93,94]. Interference occurs only when the optical paths match within the coherence length, allowing absolute surface position, optical thickness, or interface location to be extracted from the fringe signal [89,92,93,94,95]. This provides a more quantitative *Z* reference than relative focus-error methods and can be less sensitive to moderate changes in reflectivity or tilt [89,91,96]. However, real-time processing of interferometric spectra during high-speed transverse scanning requires specialised hardware, such as FPGA- or GPU-based reconstruction, and the optical architecture is correspondingly more complex and expensive [91,93,97,98].

These autofocus and distance-sensing methods address an essential but incomplete part of the 3D fabrication problem. They can stabilise the position of the focal volume relative to a surface or interface, but they do not by themselves identify the material state created inside the written voxel. In two-photon polymerisation, the written voxel is defined entirely within the bulk of a transparent photoresist by the nonlinear confinement of two-photon absorption to the focal volume—a process that has no surface analogue and that requires volumetric, not surface-referenced, process control to maintain voxel dimensions, threshold behaviour, and proximity corrections across depth. This distinction becomes critical in modern femtosecond direct writing, where the laser does not merely define morphology but can also transform local optical, electrical, chemical, structural, and ferroic properties. A written voxel may therefore be multi-featured, it can possess a modified refractive index [99], birefringence [100], conductivity [101], crystallinity [102], defect density [103], oxidation state [104], stress field [105], or nanoscale phase composition [106]. The most striking property changes include the conversion of transparent diamond into conductive graphitic or graphenic networks [107,108], laser-induced amorphous-to-crystalline transitions [109], photo-response modification in patterned 2D materials [110], direct formation of oxygen-vacancy-rich metal-oxide semiconductors [111], and ultrafast synthesis of correlated metal-oxide transition materials [14].

This multi-property character of the written voxel has direct consequences for stitching. In conventional lithography, stitching error is treated as a positional problem: a misalignment of geometric boundaries between adjacent write fields. In femtosecond direct-write, misalignment of the addressable fields can also cause discontinuities in refractive index, birefringence, stress, or crystallinity at the stitch boundary, degrading the device’s optical, electrical, or mechanical performance even when geometric alignment is achieved. A stitching strategy that achieves spatial continuity but not material-state continuity is therefore insufficient for precision photonic, quantum, or optoelectronic devices. This motivates feedback not only on position, but on the local material state itself, exactly the role that multidimensional holographic and polarisation-resolved diagnostics are designed to fill.

The resulting properties may vary laterally, axially, and through the depth of the substrate, so the fabricated structure should be regarded as an embedded heterostructure rather than as a simple surface relief or binary exposure pattern. Consequently, the 3D challenge in femtosecond lithography is not only to track the surface, but also to suppress focus drift or compensate for substrate curvature. It is to monitor and control the spatial distribution of dissimilar material states inside a volume. A discriminating measurement strategy is therefore required: one that can separate geometrical displacement from changes in refractive index, absorption, scattering, birefringence, crystallinity, conductivity, or phase composition. This motivates the use of multidimensional optical diagnostics, including holographic reconstruction, polarisation-resolved imaging, interferometric depth sensing, and high-speed transient monitoring, as components of an adaptive lithography loop rather than as post-process inspection tools.

## 5. Computational Imaging-Based Adaptive Lithography

Among the computational imaging modalities suited to in-process monitoring of femtosecond direct write, another solution to be considered is the four-polarisation (4-pol.) method [112,113]. It is characterised by acquiring images at four sequential linear polarisation orientations, the method reconstructs edge contrast and feature orientation at spatial frequencies beyond the conventional diffraction limit, in both transmission and reflection geometries. This super-resolution edge sensitivity makes 4-pol. imaging a candidate for detecting sub-voxel material transitions, such as crystallinity change or refractive index modification, without requiring physical contact with the sample. These virtues can be harnessed in monitoring and feedback, enabling adaptive correction not only of focus position but of the written property distribution itself.

The future of large-area and 3D nanofabrication is increasingly shaped by the convergence of lithography with computational imaging. While conventional lithography focuses on controlled material exposure, computational imaging provides quantitative reconstruction of optical fields and material responses, enabling adaptive, feedback-driven fabrication. In this context, holography represents a natural bridge between imaging and fabrication, as it encodes the full optical wavefront and enables reconstruction of 3D information relevant to both pattern formation and process control.

Lithography and holography share a common physical basis in diffraction and interference. Optical lithography defines structures by spatially controlling the exposure of a resist, whereas holography reconstructs the amplitude and phase of optical fields that generate these exposure distributions. This shared wave-optical foundation enables holography to extend lithographic capabilities beyond static pattern definition toward dynamic control of exposure, alignment, and material interaction. In this context, “multi-dimensional holography” refers specifically to the simultaneous or sequential exploitation of multiple physical dimensions of the optical field and the fabricated structure: the three spatial dimensions of the written volume; the temporal dimension, accessed via high-speed and time-gated imaging of transient laser–matter interaction; and the polarisation dimension, accessed through four-polarisation computational imaging schemes. By sampling the optical response under multiple polarisation states, this latter dimension provides sensitivity to sub-diffraction edge contrast and feature anisotropy. As a consequence, the reconstructed holographic signal encodes not only geometric information but also material-state variations—such as local changes in refractive index, birefringence, crystallinity, or phase composition—at sub-voxel scales. It is this multi-dimensional character—not merely 3D spatial capability—that distinguishes the proposed framework from conventional single-axis autofocus or standard holographic lithography.

A key emerging direction is the integration of computational wavefront reconstruction into fabrication workflows [114,115,116,117,118]. Rather than relying solely on pre-defined exposure conditions, computational imaging enables post-acquisition analysis of the optical field and fabricated structure, allowing adaptive correction of aberrations, alignment errors, and process variations. Such capability is particularly relevant for large-area direct write lithography, where stitching errors, focus drift, and surface non-uniformity limit pattern fidelity.

Understanding the underlying laser–material interaction during femtosecond fabrication is essential for achieving precise and reproducible pattern formation, particularly in large-area and multi-scale lithography. The interaction of ultrashort laser pulses with matter produces highly transient processes, including plasma formation, material ejection, debris generation, and bubble dynamics in surrounding media. These phenomena directly reflect the local energy deposition and interaction regime and therefore provide valuable diagnostic information for process optimisation.

**Figure 5:**
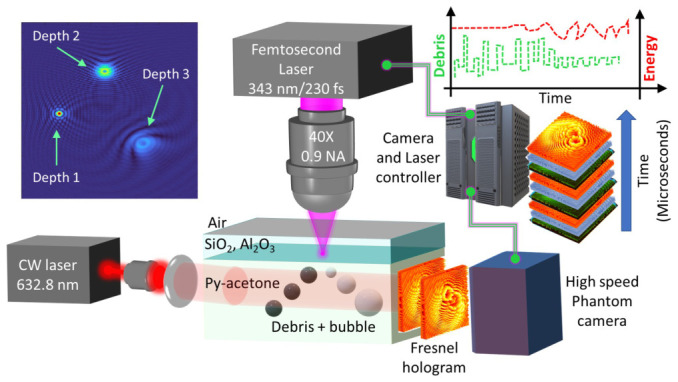
Conceptual scheme of real-time monitoring and feedback control in femtosecond laser fabrication based on characterisation of laser–material interaction dynamics. The actual fabrication without such monitoring was reported in ref. [121]. The individual sensing components of this framework have been experimentally demonstrated: Fresnel holographic depth-resolved reconstruction of transient laser–matter dynamics was reported in refs. [119,120]. The fully integrated closed-loop system represents the proposed next step.

A conceptual framework for real-time monitoring and feedback control of femtosecond fabrication is illustrated in Figure 5. During laser exposure, transient signatures such as debris ejection and bubble formation are monitored using complementary imaging modalities. Fresnel holography enables depth-resolved reconstruction of the interaction region, allowing volumetric characterisation of the evolving structure and transient features at multiple axial positions. In parallel, high-speed imaging captures time-resolved dynamics of the laser–matter interaction on microsecond time scales, providing direct observation of energy transfer processes and material response [119,120].

The spatio-temporal evolution of debris and bubble formation provides insight into local fabrication conditions, including energy deposition, ablation efficiency, and material modification mechanisms. By analysing these interaction signatures in real time, it becomes possible to identify deviations from desired processing regimes and adjust exposure parameters accordingly. Such process-aware monitoring enables adaptive control of laser writing conditions, including pulse energy, focusing conditions, and scanning parameters.

The integration of holographic imaging and high-speed characterisation establishes a computational imaging framework for feedback-controlled fabrication. In this approach, the measured interaction response is processed to infer fabrication conditions and dynamically optimise the writing process. This closed-loop strategy reduces reliance on post-process inspection and provides a pathway toward robust, adaptive, and stitch-less lithographic patterning, particularly for large-area and 3D fabrication.

Beyond sensing and feedback, holography enables a parallel mode of 3D patterning that is inherently stitch-free. Spatial light modulators (SLMs) acting as programmable phase masks can split a single femtosecond beam into hundreds or thousands of independently addressed focal spots, each writing a voxel simultaneously [122]. Digital holography-based two-photon lithography platforms have demonstrated 90 nm resolution with up to 2000 individually programmable foci and fabrication rates exceeding 2 × 10^6^ voxels/s [122]. At a larger scale, metalens-generated focal spot arrays comprising more than 120,000 cooperative foci across a 12 cm^2^ aperture and achieved throughputs exceeding 10^8^ voxels/s with sub-micrometre resolution and no stitching discontinuities with an SLM [123]. In this parallel holographic modality, stitching is eliminated not by minimising field-boundary errors but by writing the entire pattern simultaneously within the holographically defined exposure volume. The convergence of such holographic writing capability with the holographic sensing framework defines the full scope of multi-dimensional holography in lithography: exposure, alignment, and real-time process feedback all operating within the same wave-optical framework.

The practical realisation of this closed-loop framework faces several engineering challenges that must be acknowledged. Computational latency is the primary constraint: processing a Fresnel holographic reconstruction frame fast enough to update SLM patterns or pulse energy within a single scan line requires sub-millisecond computation, which currently demands GPU- or FPGA-based pipelines operating in parallel with the write head. SLM refresh rates (typically 60–1000 Hz for liquid-crystal-on-silicon devices) impose a fundamental limit on the bandwidth of adaptive wavefront correction during high-speed scanning. In multi-focus parallel writing, cross-talk between adjacent holographic foci—arising from SLM pixelation errors and higher-order diffraction—introduces non-uniformity in the voxel property distribution that is not present in single-focus sequential writing and must be characterised and corrected. These bottlenecks define the current engineering frontier and are expected to drive development in high-speed spatial light modulation, real-time holographic reconstruction algorithms, and calibration protocols over the coming decade.

More broadly, real-time characterisation of laser–material interaction represents a transition from open-loop exposure toward interaction-informed fabrication, where process dynamics guide pattern formation. Such approaches are expected to play an increasingly important role in next-generation nanofabrication systems that require high precision, scalability, and multidimensional control.

## 6. Conclusions and Outlook

For applications driven by o-Moore’s scaling and enabled by sequential direct write fabrication, the next stage of femtosecond lithography will be characterised by metrics far beyond simple feature size reduction. After approximately 25 years of rapid development in ultrashort-pulse sources, beam delivery, and laser–matter processing, the critical technological directions are now stitch-free writing, multi-beam parallelisation, adaptive beam control, and real-time correction of the writing volume. These requirements arise because direct laser writing is no longer simply a method for drawing geometrical patterns. In many material systems, the written voxel is a local material state whose properties may include refractive index, birefringence, crystallinity, conductivity, oxidation state, defect density, chemical composition, or ferroelectric order. The central hypothesis advanced here is that future femtosecond lithography must treat each voxel as a multi-property object, rather than a binary, exposed or unexposed volume. Stitch-free fabrication is consequently not only a positioning problem; it is a state-continuity problem in which adjacent voxels must preserve the intended optical, electrical, structural, and chemical properties across large areas, curved substrates, heterogeneous stacks, and buried 3D architectures.

**Figure 6:**
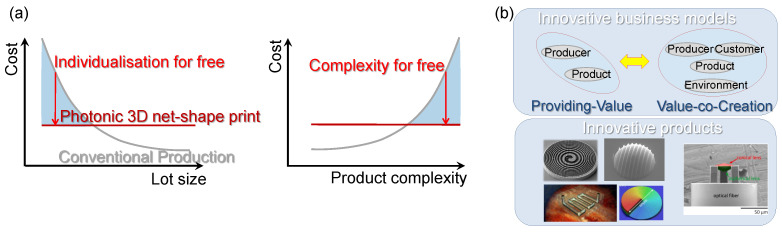
How 3D direct net-shape write prototyping can become industrially competitive in Industry 4.0. (**a**) Cost dependence on volume and complexity. (**b**) New 3D net-shape printing innovations in business and products (the model applied for Selective Laser Etching (SLE) at the Fraunhofer Institute for Laser Technology; courtesy of Dr. J. Gottmann).

This hypothesis strengthens the “Better Before Cheaper” argument. Femtosecond direct laser writing should not be judged only against projection lithography by throughput or cost per unit area, where sequential writing is intrinsically disadvantaged. Its near-term value lies in providing fabrication capabilities that parallel lithography cannot yet deliver: programmable 3D access, local material transformation, compatibility with heterogeneous substrates, rapid design iteration, and adaptive correction at the level of individual features or voxels. In this sense, the role of direct writing is to deliver better functionality before cheaper replication becomes relevant. Once a geometry, process window, or multi-property voxel state has been validated, selected parts of the process may be transferred to higher-throughput methods such as imprinting, projection exposure, or hybrid batch fabrication. However, the discovery, optimisation, and early deployment of complex 3D photonic, optoelectronic, quantum, microfluidic, and packaging architectures will continue to require the flexibility and local controllability of femtosecond direct writing. Development of 3D lithography is expected to have similarity with 3D printing, where the unique advantage value is in complexity and a minimised number of steps (hence net-shape) required for the final product, rather than relying on mass production to deliver a competitive price for the item (Figure 6). As Nobel laureate Professor Jean-Marie Lehn highlighted in his plenary talk at Imagine Nano 2015 in Bilbao [124], the future of nanotechnology lies in the complexity of developed solutions rather than solely on the pursuit of ever-finer resolution; much like the brain, which operates on a micron scale rather than purely on a nanoscale.

The future direction is therefore a transition from open-loop exposure to closed-loop, interaction-informed state lithography. Computational imaging, multidimensional holography, polarisation-resolved imaging, interferometric focus tracking, optical coherent tomography (OCT)-like depth sensing, plasma-emission diagnostics, and high-speed transient imaging should be integrated not as auxiliary inspection tools, but as components of the fabrication loop. Their task is to estimate the evolving voxel state and update pulse energy, scan speed, focus position, wavefront, polarisation, repetition rate, hatch spacing, and beam multiplexing in real time. To make this transition quantitative, future studies should report complete dose-response maps including pulse energy, average power, fluence, peak intensity, repetition rate, scan speed, pulse overlap, numerical aperture, depth, and accumulated pulse number. Threshold behaviour should be presented using normalised or logarithmic dose coordinates where appropriate, while manufacturing residuals such as line width error, edge placement error, refractive-index variation, loss, birefringence, conductivity, crystallinity, and defect density should also be reported on linear scales. The industrial integration of maskless 3D lithography will depend not merely on multi-beam or stitch-free writing, but on calibrated metrology that links in situ optical observables to ex situ material and device performance. This is the route by which femtosecond lithography can remain “better” at the stage where new architectures are invented, and eventually become “cheaper” when those architectures are stabilised and scaled.

Several practical bottlenecks must be overcome for femtosecond direct-write lithography to achieve broad industrial integration. Throughput remains the primary challenge: even with multi-beam holographic parallelisation, write speeds are orders of magnitude below those of projection lithography for large-area, low-complexity patterns. Calibration stability—particularly the long-term reproducibility of voxel properties across thermal cycles, substrate batches, and objective replacements—is a largely unsolved metrological problem that limits yield in multi-day fabrication runs. Integration with existing semiconductor workflows poses additional constraints: direct-write tools must be compatible with cleanroom environments, resist chemistries, and process control systems designed around projection lithography. Roll-to-roll or step-and-repeat hybrid approaches, in which direct write defines only the most complex or unique features while standard projection tools handle the repetitive elements, are likely to represent the most practical near-term adoption pathway.

## Data Availability

No new data were created or analysed in this study. Data sharing is not applicable to this article.
